# The Metagenomics and Metadesign of the Subways and Urban Biomes (MetaSUB) International Consortium inaugural meeting report

**DOI:** 10.1186/s40168-016-0168-z

**Published:** 2016-06-03

**Authors:** The MetaSUB International Consortium, Christopher Mason, Ebrahim Afshinnekoo, Sofia Ahsannudin, Elodie Ghedin, Timothy Read, Claire Fraser, Joel Dudley, Mark Hernandez, Christopher Bowler, Gustavo Stolovitzky, Ariel Chernonetz, Andrew Gray, Aaron Darling, Catherine Burke, Paweł P. Łabaj, Alexandra Graf, Houtan Noushmehr, s Moraes, Emmanuel Dias-Neto, Juan Ugalde, Yongli Guo, Yiming Zhou, Zhi Xie, Daisy Zheng, Hongwei Zhou, Leming Shi, Sibo Zhu, Anyi Tang, Tomislav Ivanković, Rania Siam, Nicolas Rascovan, Hugues Richard, Ingrid Lafontaine, Colin Baron, Narasimha Nedunuri, Bharath Prithiviraj, Sikander Hyat, Shaadi Mehr, Kambiz Banihashemi, Nicola Segata, Haruo Suzuki, Celia M. Alpuche Aranda, Jesus Martinez, Ayokunle Christopher Dada, Olayinka Osuolale, Folarin Oguntoyinbo, Marius Dybwad, Manuela Oliveira, Andreia Fernandes, Manuela Oliveira, Andreia Fernandes, Aspassia D. Chatziefthimiou, Salama Chaker, Dmitry Alexeev, Dmitry Chuvelev, Alex Kurilshikov, Stephan Schuster, Geoffrey H Siwo, Soojin Jang, Sung Chul Seo, Sung Ho Hwang, Stephan Ossowski, Daniela Bezdan, Klas Udekwu, Klas Udekwu, Per O. Lungjdahl, Olga Nikolayeva, Ugur Sezerman, Frank Kelly, Sarah Metrustry, Eran Elhaik, Gaston Gonnet, Lynn Schriml, Emmanuel Mongodin, Curtis Huttenhower, Jack Gilbert, Mark Hernandez, Elena Vayndorf, Martin Blaser, Eric Schadt, Jonathan Eisen, Christopher Beitel, David Hirschberg, Lynn Schriml, Emmanuel Mongodin

**Affiliations:** Dept. of Physiology and Biophysics, Weill Cornell Medicine, New York, NY 10021 USA

**Keywords:** Microbiome, Biosynthetic gene clusters, Built environment, Next-generation sequencing, Antimicrobial resistance markers

## Abstract

The Metagenomics and Metadesign of the Subways and Urban Biomes (MetaSUB) International Consortium is a novel, interdisciplinary initiative comprised of experts across many fields, including genomics, data analysis, engineering, public health, and architecture. The ultimate goal of the MetaSUB Consortium is to improve city utilization and planning through the detection, measurement, and design of metagenomics within urban environments. Although continual measures occur for temperature, air pressure, weather, and human activity, including longitudinal, cross-kingdom ecosystem dynamics can alter and improve the design of cities. The MetaSUB Consortium is aiding these efforts by developing and testing metagenomic methods and standards, including optimized methods for sample collection, DNA/RNA isolation, taxa characterization, and data visualization. The data produced by the consortium can aid city planners, public health officials, and architectural designers. In addition, the study will continue to lead to the discovery of new species, global maps of antimicrobial resistance (AMR) markers, and novel biosynthetic gene clusters (BGCs). Finally, we note that engineered metagenomic ecosystems can help enable more responsive, safer, and quantified cities.

## Introduction

In the past few years, novel work has characterized the microbiota and metagenome of urban environments and transit systems and demonstrated species-specificity to certain areas of a city, “molecular echoes” of environmental events, and even a forensic capacity for geospatial metagenomic data [1–8]. These data are especially helpful for understanding the sites of greatest points of contact between humans and the microbial world within cities, such as their subways or mass-transit systems [1–3, 7]. Indeed, how humans interact with (or acquire) new species of bacteria and other organisms depends on the environment they transit, the types of surfaces they touch, and the physical dynamics of their environment in their city. While a wide variety of methods, protocols, algorithms, and approaches for such large-scale studies are available for researchers, best practices, normalized methods, and ideal taxonomic approaches for global work are still being developed to ensure data quality and the promotion of robust data interpretation [9–12].

Since the majority of the world’s population (54 %) currently resides in cities, the use of integrative functional genomic methods to elucidate the molecular dynamics (DNA, RNA, proteins, and small molecules) and ecosystems of cities has potentially large implications for the sustainability, security, safety, and future planning of cities [13]. This includes the concept of “smart cities,” which could detect and respond to pathogens, improve water safety and treatment, and track the ever-changing metagenomic complexity of urban environments [14–17]. Indeed, by establishing a baseline genomic profile for a city, it is then possible to create differentials and density maps of organisms relevant for the built environment, such as mold and insects [18], as well as the ability to discern the impact of temperature, pressure, humidity, building materials, and other factors into the movement of organisms across a city. However, integrating the many disparate types of data generated from entire cities requires an interdisciplinary approach bringing together experts in engineering, public health, medicine, architecture, microbiology, metagenomics, bioinformatics, biochemistry, data science, functional genomics, virology, architectural design, and the built environment. Thus, in order to bridge these disciplines and work across cities with global standards and approaches, in 2015, we initiated the Metagenomics and Metadesign of Subways and Urban Biomes (MetaSUB) International Consortium.

Beyond the taxonomic classification and stratification of known and novel species that span a city, these data can be mined for other purposes. This includes characterizing novel markers for antimicrobial resistance (AMR), as well as biosynthetic gene clusters (BGCs), which can discern and validate the small molecules encoded by these organisms’ genomes and dynamically regulated transcriptomes [19, 20]. Since bacteria use small molecules to mediate microbial competition, microbial cooperation, and environment sensing and adaptation, we hypothesize that identifying the suite of small molecules produced by bacteria that are living in urban areas will reveal hidden traits of their adaptation to their successful colonization of variegated surfaces [21]. Several small molecules have been previously isolated from thermophilic and halophilic bacteria, providing a first glance of the metabolic capacity of extremophiles. These include antibacterial molecules, thought to confer a competitive advantage in harsh environments, and siderophores, which act as molecular “scavengers” of trace metals in limited conditions [22, 23]. Thus, MetaSUB’s global concerted efforts to map “urban genomes” is not only a window into urban biological systems but also a concomitant search for novel drugs, antibiotics, and small molecules that may provide new avenues for drug development and design.

## 2015 inaugural meeting of the MetaSUB Consortium

The Inaugural MetaSUB Meeting was sponsored by the Alfred P. Sloan Foundation and held on June 20, 2015, at the New York Genome Center (NYGC), following the Microbes in the City Conference on June 19, 2015, at the New York Academy of Sciences. This represented the first gathering and open meeting of the MetaSUB International Consortium. We had 30 speakers representing a wide array of expertise and disciplines, from microbiology and genomics to building/subway design and metadata collection. The meeting had 139 registrants from over 14 countries, and many speakers and attendants noted that this represented the “coming out of the shadows” of the microbes in our cities and the beginning of using these data to make cities quantified and more integrated [24, 25]. The meeting also featured a key discussion about the promises and pitfalls of metagenomics analysis, including a discussion of some of the first metagenomic data collected in NYC, Hong Kong, and Boston subways [1–3, 26].

To organize the goals of the Consortium, five working groups convened, led by five moderators. The sessions included (1) Sample Collection and Metadata led by Lynn Schriml, Ph.D., University of Maryland School of Medicine; (2) Sample Processing and Sequencing led by Daniela Bezdan, Ph.D., Center for Genomic Regulation in Spain; (3) Bioinformatics Analytics led by Brian Kidd, Ph.D., Icahn School of Medicine at Mount Sinai; (4) Visualization and Interpretation led by Elizabeth Hénaff, Ph.D., Weill Cornell Medicine; and (5) Ethical and Social Challenges led by Nathan Pearson, Ph.D., New York Genome Center. The summaries of these discussions have been outlined below and are also posted on the study’s website (www.metasub.org). The results of these working group discussions have built the foundations of MetaSUB, as each working group dealt with a key challenge the MetaSUB consortium will have to address with this global study. These working groups will evolve into committees that members of the consortium can sit on and lead. All the work by these committees will be reviewed by an external advisory board (EAB) made up of experts in the fields of bioinformatics, virology, microbiology, immunology, genomics, and mass transit. This includes Elodie Ghedin, Ph.D., New York University, Timothy Read, Ph.D., Emory University, Claire Fraser, Ph.D., University of Maryland School of Medicine, Joel Dudley, Ph.D., Icahn School of Medicine at Mount Sinai, Mark Hernandez, PE, Ph.D., University of Colorado, and Christopher Bowle, Ph.D., Institut de Biologie de l’Ecole Normale Supérieure.

## Summary of key points from working groups

### Sample collection and metadata

Any large-scale collection effort requires a detailed protocol and test of best practices, which was a key focus of the meeting. The discussion highlighted a number of challenges and suggestions related to sampling methods, standardization of protocols for data collection and processing, and validation and comparability of metadata. Also, some of the questions regarding MetaSUB collections spanned a range of unknown aspects of urban microbiomes. This ranged from the regularity of metagenomic species compositions (across time and space), the sensitivity of a surface to harboring bacteria or DNA in the context of weather, temperature, humidity, usage, and other metadata, the thresholds for persistence, the biochemical and biological functions of organisms as a function of their location, and the different methods for air vs. surface collection. The significant results of this working group are the following:

- There should be a standardized protocol for sampling across all the MetaSUB cities, reducing variability, as has been done for the FDA’s Sequencing Quality Control Consortium, the Genome in a Bottle Consortium, and the Metagenomics Standards Groups like the Earth Microbiome Project [9, 10, 27–30].
- Several series of controlled experiments should be conducted to determine what factors impact the quality of the samples, specifically, the DNA yield and potentially diversity of samples (e.g., number of passengers, humidity, air flow, temperature, sampling devices, sample storage)
- Establish a standard way to assess cleaning treatment of the different subway systems.
- Both surface-based and air sampling should be conducted in each of the city transit systems.
- The sampling protocol and metadata selection should be based on a hypothesis-driven and question-based approach that can be uniform across all cities.
- Design the most effective and efficient data collection application (“app”) that will be functional in all cities, store the metadata, upload it onto a web database, and integrate with geospatial data to create a map of collections. These include the fields of Table 1.

**Table 1:** Data fields for MetaSUB mobile data collection

Category	# of fields	Fields, with input from OSBSS metadata
MetaSUB data type	15	Soil, Superfund site, waterway, land/sea/air interface, subway, marine wild-life, synthetic ecologies, cockroach, bedbug, pigeon, rat, worm, lab mice, NYC homes, sewage
Surface composition	9	Metal, wood, plastic, ceramic, metal, leather, concrete, glass, other
Surface type	8	Kiosk, turnstile, bench, railing, handrail, garbage can, payphone, other
Surface porosity	3	Hermetically sealed, porous, absorbent
Cleaning frequency	1	Frequency per day
Cleaning type	1	Text for detergent or methods used
Human activity	3	Video, IR, and observational estimates of # of people
Air vents	3	Number of input and output vents
Subway lines	22	1, 2, 3, 4, 5, 6, 7, A, C, E, B, D, F, M, G, J, Z, L, S, N, Q R
Subway stations	468	Auto-complete from form
Subway car position	3	First car, N + l car, last car
Train ID	1	Train# 4673
Temperature	1	Range from −50°F to 15 CTF
Humidity	1	Range from 0 to 100 %
Park surfaces	9	Bench, handrailing, water fountain, slide, monkey bars, swings, trash can, lamp post, other
Audio	3	Record, play, delete
Geotag and time	1	GPS-coordinates (longitude and latitude) and time-stamp
Photograph	1	iOSor android-based

### Sample processing and sequencing

A key challenge in metagenomic studies is to obtain a representative picture of heterogeneous environmental samples and to avoid sample processing-based biases when comparing samples collected at different sites and time points. In theory, DNA isolated from a metagenomic sample should represent the biodiversity in complex populations. In reality, the quality of the information that can be generated and analyzed is highly dependent on how the samples have been collected, stored, and processed. Therefore, the goal of this working group is to (1) define standards for sample swabbing, storage, DNA extraction, sequencing library preparation and sequencing, (2) benchmark available sample processing methods, (3) survey the reproducibility of protocols at different centers, and (4) communicate defined standards to MetaSUB collaborators and the public. To this end, advantages, limitations, and potential issues of available swabbing, DNA extraction, and library preparation methods need to be investigated, and candidate methods need to be benchmarked on diverse sample types.

A main issue for sample processing is the heterogeneity of environmental samples. MetaSUB swabs will differ in DNA content and quality as well as microbiome composition, i.e., contain variable fractions of gram-negative and gram-positive bacteria, viral, fungi, and other populations of organisms. Variable susceptibility of cell structures to lytic reagents will introduce biases during DNA extraction. In addition, many microorganisms are present in the form of spores, which demonstrate high resistance to lytic practices [31]. The heterogeneous sample aggregates will range from solid to liquid, and are in most cases temperature, pH, and oxygen sensitive. Therefore, it is crucial to take parameters of the sample habitat and conditions like temperature, pH, or salinity into account for optimal selection of sample processing and library preparation methods (see Table 1 for collected data fields) or to account for introduced biases during statistical analysis of the sequencing data.

#### Sample swabbing and storage

Since cotton swabs could lead to significant contamination with cotton DNA during extraction, we first concluded that plant-based collection media would be avoided. Thus, collections should use the previously-utilized, nylon-flocked swabs (Copan Liquid Amies Elution Swabs 480C), retained in 1 ml transport medium. Minimal generation times of microorganisms range from a few minutes to several weeks [32]. Therefore, to avoid growth bias, environmental samples should be kept on ice during transportation to preserve their initial species composition. Samples are stored at−20 °C or below. Workbenches and non-sterile materials must have been cleaned with bleach and ethanol to avoid any cross-contamination.

#### DNA extraction

Two ways to extract DNA have been proposed: (1) direct extraction of DNA in situ by lysis of the bacterial cells within the sample and (2) indirect extraction by separation of bacterial cells from other organic and inorganic materials followed by DNA extraction. One of the main disadvantages of the direct extraction methods is the elevated risk of contamination with humic acids, proteins, polysaccharides, lipids, minerals, non-bacterial DNA, and minerals. Those contaminations can be difficult to remove and can inhibit chemical and enzymatic steps required for DNA processing and library preparation. On the other hand, the indirect extraction of DNA by extraction of bacterial cells from the sample likely leads to an incomplete representation or bias in content measures of bacterial species within the sample [33]. Thus, MetaSUB currently plans to use direct DNA extraction protocols, such as MoBio PowerSoil kit.

**Fig. 1:**
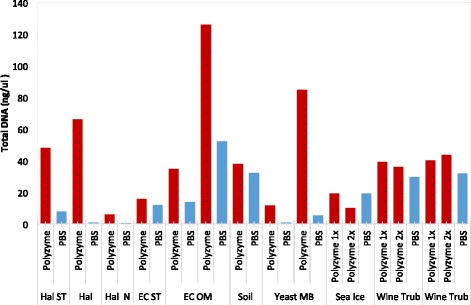
Performance of new metagenomics enzyme cocktail. We used replicate samples for a range of different extraction kits with (*red*) and without (*blue*) the polyzyme mixture (*x*-axis) and examined the yield of DNA from the extraction (*y*-axis). Samples include *Halobacillus* (*Hal*), *Escherichia coli* (*EC*), soil samples, yeast, sea ice, and a wine trub for the Omega Mullusc Kit (*OM*), Norgen Soil Kit (*N*), and the Standard CTAB-Phenol protocol (*ST*)

However, we will also compare and test various extraction protocols, combining mechanical, chemical, and enzymatic lyses steps for the several reasons. Mechanical methods like bead-beating homogenizations, sonification, vortexting, and thermal treatments like freezing-thawing or freezing-boiling tend to yield the most comprehensive access to DNA from the whole bacterial community as they allow to expose DNA from bacteria in micro-aggregates and spores. Extensive physical treatment could lead to DNA shearing resulting in fragments ranging from 600 to 12 kb, which, while not a problem for short fragment sequencing techniques (e.g., Illumina HiSeq) but would be problematic for long-read technologies (e.g., Pacific Biosciences, Oxford Nanopore MinION). Chemical cell disruption by detergents is another widely used technique. The most commonly employed chelating agents are SDS, EDTA, Chelex 100, and various Tris- and Natrium phosphate buffers. Other chemical reagents like cetyltrimethyl-ammonium bromid (CTAB) are able to remove humic acid to some extend. Humic acid contaminations are problematic since they share similar chemical and physical characteristics like DNA and co-purified humic acids also interferes with the DNA quantification, since they exhibit absorbance between 230 and 260 nm as well. Finally, enzymatic methods complement mechanical and chemical techniques by disrupting cell walls of gram-positive bacteria, which tend to be resistant to physical stress. In addition, they facilitate removal of RNA and protein contaminations, even though single-stranded and double-standed RNA viruses are an important component of the metagenomic profiles (ongoing efforts are being to made to get all of these as well). Most commonly used enzymes are lysozymes, RNase, and proteinase K (2015). Currently, members of the consortium are testing a new enzyme cocktail for DNA extraction consisting of lysozyme, mutanolysin, achromopeptidase, lysostaphin, chitinase, lyticase, and proteinase K (Fig. 1), which so far show improved yields across multiple commonly used kits for metagenomics extraction.

#### Sequencing library preparation

The current gold standard for metagenomic sequencing is based on paired-end sequencing on the Illumina HiSeq (2500 or 4000) using 100 to 150 bp paired reads. Longer reads of up to 300 bp as produced by the MiSeq increase specificity of read alignments and hence improve identification of bacterial species. However, the substantial increase in per-base cost of sequencing leads to lower depth-of-coverage and can dramatically reduce the detectability of bacterial populations contained in very small fractions. Long-read sequencing technologies (Pacific Bioscience SMRT and Oxford Nanopore MinION) promise to substantially improve classification of bacterial DNA by simplifying de novo assembly of novel species and by allowing to span complete operons and bridging long repeats with a single read. The Roche 454 platform, which has been a cornerstone of metagenomics in several studies, has not been considered here, as the technology has been discontinued. Based on these considerations, we concluded that all MetaSUB samples will be sequenced using the Illumina HiSeq platform and 150 bp paired-end reads. The application of long-read technologies will be tested on a subset of samples, and results will be benchmarked based on short read results. Finally, the inclusion of a positive control sample with known bacterial and metagenomic samples present was recommended, such as those from the Genome Reference Consortium (GRC) and US National Institute of Standards and Technology (NIST).

### Bioinformatics analytics

With the advent of citizen science, crowdsourcing, and participatory international coordination of sampling, the ability to collect large metagenomic datasets from our surroundings is no longer the limiting factor in scientific discovery and exploration of the microbial landscape in urban environments [35]. As the tide has shifted, key questions about ideal methods to analyze and process the data have become paramount, and multiple analytical challenges have arisen for computing, processing, and sharing of metagenomic data [12]. Addressing these analytical challenges has implications for how we understand and interpret the diversity and complexity of urban biomes. The bioinformatics working group discussed current analytical challenges facing the consortium and suggests protocol adaptations as technologies improve. What emerged from the discussion were four themes covering (1) standards, (2) reproducibility, (3) open-access/data sharing, and (4) innovation. The central goal of the bioinformatics working group is to build on these themes over time, refining the methods, because as it currently stands, there is not a definitive set of guidelines for many of these challenges.

#### Sample standardization for benchmarking analytical tools and interpreting results

A key challenge in analyzing metagenomic sequences from urban environments is how to deal with potential novelty and sequence diversity. Metagenomic sequencing provides an unprecedented wealth of data, and probing the urban biome pushes the frontiers of our knowledge and understanding of microbes. It is thus critical to have empirical and computational standards to delineate the technical issues from true discoveries. An empirical way to address this challenge is to extraneously introduce standard control samples that have been well characterized to help interpret findings and place discoveries in context. Another approach is to generate reference data sets from various sequencing technologies that bioinformaticians and developers can use for testing and benchmarking [34]. These reference sequence sets provide ideal test cases for understanding technical issues with sequencing data or algorithms (given the known proportions of various bacteria) and supply useful benchmarks for consortium members during the development of new tools [1]. More importantly, these references serve as standards for developing clear metrics on how to evaluate and interpret results from metagenomic analyses from large numbers of people [35].

#### Data processing and reproducibility

The massive scale and volume of metagenomic data generated in studies of the urban biome exceeds our ability to conduct manual processing and quality assurance. Computational processing can alleviate this bottleneck, and it is important to develop clear quality control metrics for each link in the analytical chain (data QC, post-sequencing trimming, alignment, assembly, phylogenetics, summary statistics). As sample preparation and processing strongly influence what information can be extracted and analyzed, it is important to have strong collaborations between the computational biologists who develop the computational tools and the core facilities or labs that create the libraries and process samples for sequencing, as well as methods to detect, and correct, for batch effects [36].

Code sharing and transparency are important features of reproducibility, and open source tools such as R and Bioconductor exist for creating processing pipelines. It is important to create transparent workflows that can be cloned and deployed on remote machines so the analyses can be reproduced with minimal effort [37]. Furthermore, electronic notebooks with protocols can be linked with publications. Having version control or Docker-style tracking encourages collaboration and enables best practices to spread through the community of developers and scientists. Other large-scale consortiums such as The Cancer Genome Atlas (TCGA) and Human Microbiome Project (HMP) have successfully navigated these issues and provided a model for creating accessible data portals with community-based tools [38, 39]. In this age of abundant computing and storage, data provenance and transparency are critical for developing robust and useful methods that enable innovation while maintaining scientific rigor.

#### Data sharing and common formats

Collecting samples and generating data can be an expensive effort, yet these data sets are rich and can be leveraged when others have access to data. As a community, we want to encourage open collaboration and provide incentives for researchers to share their published data in a common format that facilitates interoperability (e.g., SAGE, HMP guidelines). We can better understand how microarray technology has matured and the data warehouses that have sprung up around the developing technology. Central clearing houses like the Gene Expression Omnibus (GEO) and European Genome-phenome Archive (EGA) include standard data fields and associated metadata that are compliant with Minimum Information About a Microarray Experiment (MIAME) guidelines [40–42]. These resources have accelerated research and collaborations by providing accessible data sets for developing novel methods and addressing new scientific questions, which are linked with the original contribution [43]. Additionally, the analysis of public data has generated many new insights and hypotheses that would not have been identified or proposed otherwise [44]. Ideally, these data sharing portals offer ways to link new insights and results back to their original source. These data warehouses establish a strong foundation for other scientists, citizens, and policy makers to develop new research strategies based on the accumulated knowledge.

#### Innovation

Technological and computational innovations will continue to define and drive investigations of urban biomes across all MetaSUB sites (Table 2). These advances create an apparent tension between being the cutting edge where analyses and conclusions are more fluid, and well-established processes that are robust and strongly supported. It is crucial to distinguish between these two modes and the computational tools that underpin them. We want to encourage the development of novel methods and work toward best practices that result in accepted pipelines that serve as a strong foundation for scientific discovery.

**Table 2:** Hub laboratories of the MetaSUB International Consortium

	City details		Site principal investigator			
Site	City	Country	Department	University/institute	Contact pis	Email
1	Buenos Aires	Argentina	1. Genetics and Genomic Sciences; 2 Computational Biology Center; 3. Departamento de Fisica	1. Icahn School of Medicine at Mount Sinai; 2 IBM; 3. University of Buenos Aires	Gustavo Stolovitzky^1,2^/Ariel Chernonetz^3^	gustavo@us.ibm.com/achernomoretz@leloir.org.ar
2	Sydney	Australia	Computational Metagenomics	University of Technology	Aaron Darling/Catherine Burke	aaron.darling@uts.edu.au/Catherine.Burke@uts.edu.au
3	Vienna	Austria	Bioinformatics/Bioinformatics	Boku University Vienna/University of Applied Sciences	Paweł P. Łabaj/Alexandra Graf	pawel.labaj@boku.ac.at/alexandra.graf@fh-campuswien.ac.at
4	Ribeirão Preto	Brazil	Department of Genetics, Laboratory of Epigenomics and Bioinformatics	University of Sao Paolo	Houtan Noushmehr	houtan@usp.br
5	Rio Da Janeiro	Brazil	Oswaldo Cruz Institute	FIOCRUZ	Milton Ozorio Moraes	milton.moraes@fiocruz.br
6	São Paulo	Brazil	Medical Genomics	AC Camargo Cancer Center	Emmanuel Dias-Neto	emmanuel@cipe.accamargo.org.br
7	Santiago	Chile		Universidad del Desarrollo	Juan Ugalde	jugalde@udd.cl
8	Beijing	China	Beijing Children's Hospital/Translational Bioinformatics Research Institute	Capital Medical University/Capitalbio Corp	Yongli Guo/Yiming Zhou	ylgyongliguo@163.com/yimingzhou@capitalbio.com
9	Guangzhou	China	1.State Key Lab of Ophthalmology, Guangdong Provincial Key Lab of Ophthalmology and Visual Science, Zhognshan ophthalmic Center, Center for Precision Medicine, School of Public Health; 2. Department of Environmental Health; 3. Division of Laboratory Medicine at Zhujiang Hospital	1. Sun Yat-sen University; 2.Southern Medical University	Zhi Xie1,1/Daisy Zheng2,2/Hongwei Zhou2,3	xiezhi@gmail.com/180553957@qq.com/811807859@qq.com
10	Hong Kong	China	School of Energy and Environment	City University of Hong Kong	Patrick K.H. Lee	patrick.kh.lee@cityu.edu.hk
11	Shanghai	China	School of Life Science	Fudan University	Leming Shi/Sibo Zhu/Anyi Tang	lemingshi@fudan.edu.cn/sibozhu@fudan.edu.cn/491269854@qq.com
12	Bogota	Colombia	Molecular Genetics	Corporación Corpogen	Carlos A. Ruiz-Perez/Maria M. Zambrano	cruiz_perez@hotmail.com/mzambrano@corpogen.org
13	Zagreb	Croatia	Department of Microbiology	University of Zagreb	Tomislav Ivanković	tomislav.ivankovic@biol.pmf.hr
14	Cairo	Egypt	Department of Biology	American University of Cairo	Rania Siam	rsiam@aucegypt.edu
15	Marseille	France	Department of Virology	Aix-Marseille University	Nicolas Rascovan	nicorasco@gmail.com
16	Paris	France	Laboratory of Computational and Quantitative Biology	Sorbonne Universite's, University Pierre et Marie Curie Univ. Paris 06, CNRS, Institut de Biologie Paris-Seine	Hugues Richard/Ingrid Lafontaine	hugues.richard@upmc.fr/ingrid.lafontaine@upmc.fr
17	Berlin	Germany	Public Health	Robert Koch Institute	Lothar H. Wieler/Torsten Semmler	wielerlh@rki.de/SemmlerT@rki.de
18	Hyderabad	India	Department of Biotechnology and Bioinformatics	University of Hyderabad/Noble Foundation/ClonzBio Tech	Niyaz Ahmed/Bharath Prithiviraj/Narasimha Nedunuri	ahmed.nizi@gmail.com/bharath.prithiviraj@gmail.com/narasimha.nedunuri@clonzbio.com
19	New Delhi	India	Computational Biology	Memorial Sloan Kettering	Sikander Hyat	hayat221@gmail.com
20	Tehran	Iran	Ecology/Medical Sciences	American Museum of Natural History/Ministry of Science	Shaadi Mehr/Kambiz Banihashemi	smehr@amnh.org/kbanihashemi@yahoo.com
21	Rome	Italy	Molecular Biology Section	Army Medical and Veterinary Research Center	Florigio Lista/Anna Anselmo	romano.lista@gmail.com/annanselm@gmail.com
22	Sendai	Japan	Institute for Advanced Biosciences	Keio University	Haruo Suzuki	haruo@sfc.keio.ac.jp
23	Tokyo	Japan	Institute for Advanced Biosciences	Keio University	Haruo Suzuki	haruo@sfc.keio.ac.jp
24	Mexico City	Mexico	National Institute of Public Health	IANPHI Mexico Secretariat	Celia M. Alpuche Aranda/Jesus Martinez	celia.alpuche@insp.mx/jmbarnet@insp.mx
25	Auckland City	New Zealand	Environmental Research Institute	Univeristy of Waikato	Ayokunle Christopher Dada	cdada@waikato.ac.nz
26	Lagos	Nigeria	Microbiology	University of Lagos	Folarin Oguntoyinbo	foguntoyinbo@unilag.edu.ng
27	Oslo	Norway	Protection and Societal Security Division	Norwegian Defense Research Establishment FFI	Marius Dybwad	marius.dybwad@ffi.no
28	Lisbon	Portugal	Department of Biology, i3S Population Genetics and Evolution Group	University of Porto	Manuela Oliveira/Andreia Fernandes	manuelao@ipatimup.pt/afernandes@ipatimup.pt
29	Porto	Portugal	Department of Biology, i3S Population Genetics and Evolution Group	University of Porto	Manuela Oliveira/Andreia Fernandes	manuelao@ipatimup.pt/afernandes@ipatimup.pt
30	Doha	Qatar	Ecology/Medicine	Weill Cornell Medical College - Qatar	Aspassia D. Chatziefthimiou/Salama Chaker	asc2006@qatar-med.cornell.edu/salama.b.chaker@gmail.com
31	Moscow	Russia	Bioinformatics	Moscow Institute of Physics and Technology, Institutskii Per. 9, Moscow Region, Dolgoprudny 141700, Russia	Dmitry Alexeev/Dmitry Chuvelev	alexeev@knomics.ru/dch@knomics.ru
32	Singapore	Singapore	Biochemistry and Molecular Biology	Pennsylvania State University	Stephan Schuster	scschuster@ntu.edu.sg
33	Johannesburg	South Africa	Watson/Research	IBM	Geoffrey H Siwo	ghsiwo@us.ibm.com
34	Seoul	South Korea	Microbiology/Institute for Allergy and Immunology/Cancer Risk Appraisal & Prevention Branch	Institut Pasteur Korea/Korea University College of Medicine/National Cancer Center	Soojin Jang/Sung Chul Seo/Sung Ho Hwang	soojin.jang@ip-korea.org/sungchul_seo@korea.ac.kr/9954074@daum.net
35	Barcelona	Spain	Genomic and Epigenomic Variation	1. Center for Genomic Regulation (CRG), The Barcelona Institute of Science and Technology, Dr. Aiguader 88, Barcelona 08003, Spain 2. Universitate Pompeu Fabra (UPF), Barcelona, Spain	Stephan Ossowski1,2/Daniela Bezdan1,2	Stephan.Ossowski@crg.eu/bezdan.daniela@googlemail.com
36	Stockholm	Sweden	Department of Molecular Biosciences, The Wenner-Gren Institute	Stockholm University	Klas Udekwu/Per O. Lungjdahl	klas.udekwu@su.se/per.ljungdahl@su.se
37	Zurich	Switzerland	Institute of Molecular Life Sciences	University of Zurich	Olga Nikolayeva	olga.nikolayeva@gmail.com
38	Izmir	Turkey	Department of Biostatistics and Medical Informatics	Acibadem University	Ugur Sezerman	sezermanu@gmail.com
39	Sheffield	UK	Department of Animal & Plant Sciences	University of Sheffield	Eran Elhaik	e.elhaik@sheffield.ac.uk
40	Montevideo	Uruguay	Genetics	ETH Zurich	Gaston Gonnet	gonnet@ethz.ch
41	Baltimore	USA	Institute for Genome Sciences	University of Maryland School of Medicine	Emmanuel Mongodin	emongodin@som.umaryland.edu
42	Boston	USA	Biostatistics	Harvard T.H. Chan School of Public Health	Curtis Huttenhower	chuttenh@hsph.harvard.edu
43	Chicago	USA	Microbial Ecology	Argonne National Laboratory	Jack Gilbert	gilbertjack@uchicago.edu
44	Denver	USA	Mechanical Engineering	University of Colorado	Mark Hernandez	mark.hernandez@colorado.edu
45	Fairbanks	USA	Institute of Arctic Biology	University of Alaska Fairbanks	Elena Vayndorf	elena.vayndorf@alaska.edu
46	New York City	USA	Physiology and Biophysics	Weill Cornell Medicine	Christopher Mason	chm2042@med.cornell.edu
47	Sacramento	USA	Department of Interdisciplinary Arts and Sciences	UC Davis	Jonathan Eisen	jonathan.eisen@gmail.com
48	San Francisco	USA	Department of Interdisciplinary Arts and Sciences	University of California, Davis	Christopher Beitel	chris.w.beitel@gmail.com
49	Seattle	USA	Department of Genetics and Genomics	University of Washington	David Hirschberg	dhberg@uw.edu
50	Washington DC	USA	Institute for Genome Sciences	University of Maryland School of Medicine	Lynn Schriml	lschriml@som.umaryland.edu
51	London	UK	Department of Twin Research	Kings College London	Frank Kelly/Sarah Metrustry	frank.kelly@kcl.ac.uk/sarah.metrustry@kcl.ac.uk

We show the city, country, site of collaboration (university, company, or government agency), principal investigator (PI), and the number of riders per year in the targeted mass-transit system. This includes the top busiest subways in the world, except for Moscow (still recruiting PI)

### Data visualization and interpretation

**Fig. 2:**
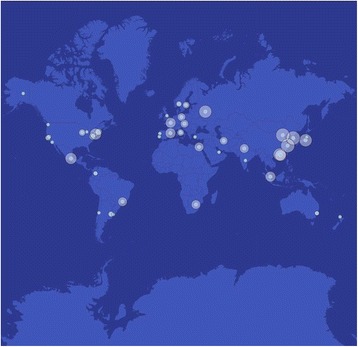
Map of active MetaSUB sites. We have shown all the sites of the MetaSUB International Consortium that are collecting. The sizes of the circles are proportional to the number of riders per year on the subway or mass-transit system

Visualization and interpretation are some of the most challenging aspects of a study this large and global. Thus, the working group outlined the goals of the consortium according to three main areas. First, there is a need to design systems of data visualization for data exploration, so that any user of the web site or resources can rapidly learn from and utilize the data [1]. Second, there must be a clear outline of the consortium organization (Fig. 2), including an ability to look at results, metadata, and milestones for each city. Third, there is a need for communicating results, collaboration, publications, and the status of outreach and citizen science efforts. This will continue to use the components of web sites, online forums, and social media such as Twitter, Facebook, and Instagram.

Each of these categories holds its own challenges and specifications, for example, visualizations for data exploration need to be much denser in information than for publication where only the information relevant to the message needs to be presented. Visualizations for outreach need to be friendly and easy to understand by non-scientists and laypeople. The medium available also influences design choices: figures designed for print media have limitations that the web does not, and we have already piloted a cross-kingdom browser for urban metagenomics (www.pathomap.org/map) [1]. In addition to visualizing scientific data, we will use visual representations to aid in the coordination and organization of the consortium, e.g., metadata regarding the number of samples collected and processed in each site. Finally, the kind of data will dictate the design of the visualizations. Such data include metadata taxa present (phylogenetic relationships and abundance), metabolic pathways, functional annotations, geospatial relationships, and time-lapse data. Finally, metadata outlined in Table 1 will also be integrated into the design of these visuals, since the metadata from a study can readily become the raw data for a follow-up study.

### Ethical, social, and legal challenges

Since the MetaSUB Consortium is a public, transparent, and open consortium that aims to characterize and discover the microbial sides of the cities in which we live, transparency is an important principle during the process of urban biome discovery, hands-on education, and city planning. Therefore, all meeting minutes, talk slides, and group listserv correspondences are posted in public archives and also on the Consortium website. Also, any grant dollars, donations, and corporate sponsorship are listed and detailed publicly as well.

Nonetheless, there are several critical ethical and social challenges that must be addressed. First, the collection of samples must be done in a transparent and assuring fashion, and work from the first studies included business cards to hand out to citizens on the street for when they had questions. Interactions from the public ranged from curiosity and extreme interest about the project to confusion of what would be found. In general, because the first data sets have shown a predominance of harmless and commensal bacteria, it is important to note the data-based assurance to the public safety and trust in public transportation. Nonetheless, there have been lessons learned from the “cautionary tale” of DNA found in NYC metagenomic data sets [12], wherein fragments of DNA that matched a pathogen must be put into the context of virulence markers and also in the context of likelihood of the samples being present. Finally, these first urban metagenome reports also show that the collection, interpretation, and release of such public data represent an extremely serious responsibility for the scientists reporting and interpreting these sensitive data.

Also, consideration of other logistical challenges related to the interpretation and release of the data and analysis are required, regarding city, transit, and health authorities in each city. Some cities may wait until data are published before deciding to comment, but nonetheless, all data and manuscripts should be shared with city officials beforehand, and this has been the standard applied thus far [1]. Also, three new guidelines have been implemented as part of MetaSUB: all data and sequences collected will be given to the local authorities for a “Right to First Review,” before any publication or presentation of these results to the public, due to the potential sensitivity of some of the species that may be discovered. Protocols will follow internationally recognized standards for quality control and sequencing rigor from the US Food and Drug Administration’s (FDA) Sequencing Quality Control Consortium (SEQC) and the Earth Microbiome Project (EMP) as outlined above. Any species discovered that are germane to bioterrorism or public health will be turned over to public health officials first and not reported without independent validation.

**Fig. 3:**
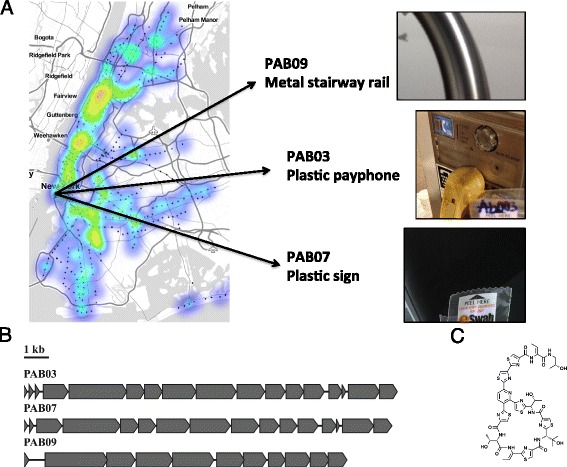
Discovery of biosynthetic gene clusters from surface-based metagenomics sampling. Plotting the density of organisms across the city shows many diverse sites from which new biology could be discovered *red* to *purple* from highest to lowest); plotted here is the *Pseudomonas* genus, and examples of three samples from the NYC PathoMap study: PAB009 (stairway railing), PAB03 (payphone), and PAB07 (sign). **b** Three predicted BGCs discovered in the corresponding samples shown in **a**. Interestingly, these three BGCs are predicted to encode known and novel small molecules of the thiopeptide/thiocillin class of antibiotics.

Finally, the ability to “mine” the metagenomic biological data for new drugs, small molecules, and antibiotics brings additional possibilities for innovation, but also complications (Fig. 3). Since each country has their own guidelines surrounding intellectual property (IP), ownership of biological data, and also the regulations around “bio-prospecting,” care must be taken to ensure that national and international guidelines for collection are met. Most current legislation around the world define “prospecting” as the collection of samples and removal from the country of origin but likely do not apply to the ability to predict the unique molecules of each country from sequence data alone. To ensure that data accessibility and attribution is maintained, and to avoid the issues with rampant patenting of nucleic acids [45], we are posting data from the consortium and ensuring BGC first-pass detection as a component of standard QC for each sample.

## Study design and goals

The final part of the meeting was to define the goals of the MetaSUB consortium, which is now planned for at least five years (2016–2020) and rooted in five core areas: collection, analysis, design, standards, and education.

### Collection

Establishing a coordinated, global data collection is slated to begin on June 21, 2016, to match and parallel the Global Ocean Sampling Day (OSD) [46, 47]. The will begin the seasonal of cities around the world for the next five years, matching at least the once-a-year frequency of (OSD), but each season if possible for each city. Notably, this time frame overlaps both the Brazilian and Japanese Olympics, generating the profile of a city’s “olympiome,” representing a first-ever sampling of cities before, during, and after a global human migration event. Sampling will be done to include: air in public parks, surfaces in subway or transit system kiosks, park water fountains, and adjacent ocean water (through OSD). Also, a subset of 50 samples will undergo some single-cell and cross-linked read capture (Hi-C), and long-read sequencing for improved species resolution. Sampling will focus on areas of mass transit, but other areas throughout the city will be considered in order to paint a clearer molecular portrait of the city and explore potential networks and feedback mechanisms that may exist.

### Analysis

There will be ongoing work for testing, sharing, and advancing computational methods. Also, we will link to and curate a global database of detected BGCs as well as antimicrobial resistance (AMR) markers. We will also use rarefaction plots and Shannon diversity indices to create cross-kingdom (plant, animal, bacterial, viral) measures of diversity between climates and cities. Finally, we will look for any evidence of horizontal gene transfer (HGT) in the samples when comparing to newly sequenced genomes from local areas.

### Design

These methods of collection that characterize many types of surfaces may have an impact on future designs and types of transit systems. There, collections include samples from many types of surfaces, including plastic, cloth, metal, ceramic, glass, and stone. In addition, we will collect metadata about temperature, humidity, volatile organic carbons (VOCs), air components, and other environmental parameters. A long-term goal of the consortium would be to design surfaces to enhance the “good bacteria” present such that they could out-complete the “bad bacteria” and make the surfaces better for human occupancy and transit.

### Standards

By deploying and testing DNA and bioinformatics standards, we will help improve methods in the field of metagenomics. Specifically, we will continue to use samples with known proportions of species for in silico measurement and testing of algorithms [1]. Also, we will use Genome Reference Consortium (GRC) and US National Institute of Standards and Technology (NIST) standards for future testing of sequencing methods. Finally, we will plan to develop synthetic oligonucleotides for positive controls during sampling to address the question of DNA/RNA bias during collection.

### Education

Using our methods for outreach, education, and hands-on training is one of the key components of the consortium. We have already engaged hundreds of students in cities associated with the MetaSUB Consortium study, and we intend to maintain this educational component. This will include some citizen science outreach for high school, college, graduate, and medical students, as well as credits for a related course (microbiology, ecology, genetics, genomics) during the sampling expeditions (“swabventure”). Also, we have started a study abroad and lab exchange program so that members of the Consortium can visit each other’s labs and sites to learn about genomics, informatics, or architecture. Indeed, we already have three artists in residence for the Consortium, all of whom work to visualize the microscopic and metagenomic world around us. Finally, we will build a program to enable a certificate of molecular microscopy, ideally as a free, online course for people to take in their own country.

### Community outreach

Along with the educational goals, MetaSUB seeks to interact with local communities, teaching others to explore the microbiome that lives in us, on us, and all around us [46]. We believe in the freedom of information and feel that citizens are entitled to know about the environment in which they live. We encourage citizens to propose certain sites to be profiled, as well as encourage their involvement in the sampling process. Our Global City Sampling Day (CSD) will be driven not only by scientists in the consortium but open to all citizens interested in exploring the molecular microbial and metagenomic dynamics of their cities and oceans (with OSD). We also feel that it is important to provide easy access to the data collected in a way that enables meaningful interpretations by the general public. We hope that residents will have a role in disseminating and discussing the results and that we will provide an additional metric with which to understand and explore our urban environment.

## Conclusion

Working together, we are building an unprecedented, global metagenomics dataset and molecular portrait of the urban microbiomes that we all share. Our collective efforts aim to help current and future work in city planning, urban design and architecture, transit systems, public health, ecological studies, genome technologies, and improved understanding of cities. We aim to use the lessons of the preliminary studies to highlight the richness of the microbial ecosystems of cities, train new students in best practices and methods for metagenomics and microbiome analysis, and ensure the greatest utility and benefit of these data. These data will also provide a novel resource to discover new biochemical pathways, sources of antimicrobial resistance, new methods of metagenomic design, and new antibiotics that are created by the ecosystem of microbes that have evolved to live among us (and we among them).

## Abbreviations

AMR: antimicrobial resistance
BGCs: biosynthetic gene clusters
CTSC: Clinical and Translational Science Center
EAB: external advisory board
EGA: European Genome-phenome Archive
EMP: Earth Microbiome Project
FDA: Food and Drug Administration
GEO: Gene Expression Omnibus
GRC: Genome Reference Consortium
HGT: horizontal gene transfer
HMP: Human Microbiome Project
MetaSUB: Metagenomics and Metadesign of Subways and Urban Biomes
MIAME: Minimum Information About a Microarray Experiment
NIST: National Institute of Standards and Technology
NYC: New York City
NYGC: New York Genome Center
OSBSS: open source building science sensors
PI: principal investigator
SAGE: SAGE Bionetworks
SEQC: Sequencing Quality Control Consortium
TCGA: The Cancer Genome Atlas
VOCs: volatile organic carbons
