# Erratum to: The Metagenomics and Metadesign of the Subways and Urban Biomes (MetaSUB) International Consortium Inaugural Meeting Report

**DOI:** 10.1186/s40168-016-0184-z

**Published:** 2016-08-18

**Authors:** The MetaSUB International Consortium

**Affiliations:** Department of Physiology and Biophysics, Weill Cornell Medicine, New York, NY 10021 USA

## Erratum

During pre-publication proofs, some final changes were not included in the manuscript and we also had several sites that joined right before the global city sampling day (CSD) that should be included. These have now been updated in the original paper [1] and the updates are listed below:

1. Rename the section “MetaSUB City Principal Investigators” to “MetaSUB Consortium Members”
  The following names should be included:
  Ken McGrath and Leanne McGrath (Brisbane, Australia)
  Andrew Gray (Melbourne, Australia)
  Olayinka Osuolale (Ilorin, Nigeria)
  Nicola Segata (Trenton, Italy)
  Silvia Fillo (Rome, Italy)
  Gregorio Iraola (Montevideo, Uruguay)
  Yiming Zhou (Beijing, China)
  Yujun Chang (Beijing, China)
  Yang Li (Beijing, China)
  Yuanting Zhend (Shanghai, China)
  Wanwan Hou (Shanghai, China)
  Adan Ramirez (Bogota, Colombia)
  Martha Cepeda (Bogota, Colombia)
  Christelle Desnues (Marseille, France)
  Nicolas Rascovan (Marseille, France)
  Amged Ouf (Cairo, Egypt)
  Colin Baron (Düsseldorf, Germany)
  Niranjan Nagarajan (Singapore)
  Danilo Ercolini (Naples, Italy)
  Wayne Menary (Lima, Peru)
  Scott Tighe (Vermont, USA)
  Mohamed Donia (Princeton, USA)
  Shawn Levy (Huntsville, USA)
  Joseph Benito (Huntsville, USA)
  Angela Jones (Huntsville, USA)
2. In the section “The following authors contributed to this manuscript,” the following name should be added:
  George Yeh, Millipore Sigma
3. Some of the affiliations in the published version of Table 2 were inaccurate. Please see the corrections below:

**Table:** 

**Site**	**City**	**Country**	**Department**	**University/institute**	**Contact PIs**	**Email**
9	Guangzhou	China	1. Zhongshan ophthalmic Center, Center for Precision Medicine; 2. Department of Environmental Health; 3. Division of Laboratory Medicine at Zhujiang Hospital	1. Sun Yat-sen University; 2.Southern Medical University	Zhi Xie1,1/Daisy Zheng2,2/Hongwei Zhou2,3	xiezhi@gmail.com/180553957@qq.com/811807859@qq.com
25	Auckland, Hamilton and Rotorua	New Zealand	Environmental Research Institute	Univeristy of Waikato	Ayokunle Christopher Dada	cdada@waikato.ac.nz
33	Johannesburg	South Africa	Data Driven Healthcare	IBM Research-Africa	Geoffrey H Siwo	gsiwo@za.ibm.com
40	Montevideo	Uruguay	Informatik	ETH Zurich	Gaston Gonnet	gonnet@ethz.ch
47	Sacramento	USA	Department of Ecology and Evolution	UC Davis	Jonathan Eisen	jonathan.eisen@gmail.com
4. The following errors in the manuscript should be corrected:
  - In “Website Curator” Sofia Ahsanuddin’s name is incorrectly spelled as Ahsannudin
  - PAGE 3: Table 1 row 2, Surface composition category, the word "metal" was repeated twice.
  - For Ayokunle Christopher Dada’s affiliation, Auckland City should be Auckland, Hamilton and Rotorua (New Zealand).
  - In the “Inaugural MetaSUB International Meeting Speakers” section, Russel Neches should be “Russell Neches”
  - In the section *DNA extraction*, Proteinase K is not in the Polyzyme mixture and therefore the sentence should read: ‘Currently, members of the consortium are testing a newenzyme cocktail for DNA extraction consisting of lysozyme,mutanolysin, achromopeptidase, lysostaphin, chitinase, and lyticase’.
  - Elena M. Vayndorf is the correct full name for Dr. Vayndorf.
  - The legend of Figure 1 should read, “Compliments of the ABRF Metagenomics Research Group”
  - Table 2 heading should change from “Contact pis” to “Contact PIs”
  - Emails for PIs of Doha are a.chatziefthimiou@richenvironments.com/salama.b.chaker@gmail.com
  - Dr. Niyaz Ahmed should be removed from the “MetaSUB Consortium Members” list.
5. Acknowledgements
  a. This work was also supported in part by the National High Technology Research and Development Program of China (2015AA020104), the National Natural Science Foundation of China (31471239) and the 111 Project (B13016).
